# Molecular Cloning and Characterization of Growth Factor Receptor Bound-Protein in *Clonorchis sinensis*

**DOI:** 10.1371/journal.pone.0085577

**Published:** 2014-01-16

**Authors:** Xuelian Bai, Ji-Yun Lee, Tae Im Kim, Fuhong Dai, Tae-Jin Lee, Sung-Jong Hong

**Affiliations:** 1 Department of Medical Environmental Biology, Chung-Ang University College of Medicine, Dongjak-gu, Seoul, Republic of Korea; 2 Department of Pathology, Chung-Ang Univesity College of Medicine, Dongjak-gu, Seoul, Republic of Korea; Hungarian Academy of Sciences, Hungary

## Abstract

**Background:**

*Clonorchis sinensis* causes clonorchiasis, a potentially serious disease. Growth factor receptor-bound protein 2 (Grb2) is a cytosolic protein conserved among animals and plays roles in cellular functions such as meiosis, organogenesis and energy metabolism. In the present study, we report first molecular characters of growth factor receptor bound-protein (CsGrb2) from *C. sinensis* as counter part of Grb2 from animals and its possible functions in development and organogenesis of *C. sinensis*.

**Methodology/Principal Findings:**

A CsGrb2 cDNA clone retrieved from the *C. sinensis* transcriptome encoded a polypeptide with a SH3-SH2-SH3 structure. Recombinant CsGrb2 was bacterially produced and purified to homogeneity. Native CsGrb2 with estimated molecular weight was identified from *C. sinensis* adult extract by western blotting using a mouse immune serum to recombinant CsGrb2. CsGrb2 transcripts was more abundant in the metacercariae than in the adults. Immunohistochemical staining showed that CsGrb2 was localized to the suckers, mesenchymal tissues, sperms in seminal receptacle and ovary in the adults, and abundantly expressed in most organs of the metacercariae. Recombinant CsGrb2 was evaluated to be little useful as a serodiagnostic reagent for *C. sinesis* human infections.

**Conclusion:**

Grb2 protein found in *C. sinensis* was conserved among animals and suggested to play a role in the organogenesis, energy metabolism and mitotic spermatogenesis of *C. sinensis*. These findings from *C. sinensis* provide wider understanding on diverse function of Grb2 in lower animals such as platyhelminths.

## Introduction

*Clonorchiasis sinensis* (*C. sinensis*) presents a serious health problem that is epidemic in East Asian countries, such as, China, Korea, Vietnam and Thailand. It has been reported that about 35 million people were infected worldwide [1]. Mammals are the final hosts of *C. sinensis* and are infected by consuming raw or uncooked freshwater fish containing metacercariae. After being swallowed, metacercariae pass through stomach to the duodenum, and then the newly excysted juveniles migrate to the intrahepatic bile duct where they develop to adult worms [2]. *C. sinensis* causes pathological changes in the biliary tree, such as hyperplasia and inflammation of biliary mucosa, periductal fibrosis and ductal wall thickening. Patients with clonorchiasis experience epigastric pain, indigestion, abdominal distension, diarrhea, edema, weight loss, and hepatomegaly. Furthermore, *C. sinensis* has been classified as a biological carcinogen by the International Agency for Research on Cancer due to its association with cholangiocarcinoma [3].

During its growth from the metacercarial to the adult form, many genes might be expressed and contribute to organogenesis. Growth factor receptor-bound protein 2 (Grb2) is a protein that is present in cytoplasm, and is composed of one SH2 domain flanked by two SH3 domains. As an adaptor protein in signal transduction pathways, Grb2 is involved in many cellular functions and pathological processes. Phosphotyrosine residues in activated receptor tyrosine kinase and adaptors such as Shc and FRS-2 are recognized by SH2 domain in Grb2. The SH2 domain of Grb2 also recognizes and binds to proline-rich motifs in target proteins such as Sos [4], [5]. For example, EGFR in the cell membrane dimerizes upon ligand (EGF) binding, and this event leads to phosphorylations of receptor tyrosines and the binding of the SH2 domain of Grb2 to EGFR. The SH3 domains then recognize and bind to their target proteins [6]. As a result, Ras is activated by Grb2/Sos complex in response to growth factors in many types of cells, such as fibroblasts, neural cells and lymphotes. Furthermore, Grb2 has been identified in diverse organisms. In *C. elegans*, Sem-5 homologous with Grb2 was found to be involved in the signal transduction pathway that controls vulva development and sex myoblast migration. In mice, Grb2 is present in many organs and participates in organogenesis and development. Grb2 also promotes the re-initiation of meiosis in *Xenopus* oocytes. In addition, Grb2 has even been reported to be associated with carcinogenesis [7], [8], [9].

However, no report has been issued about the molecular character and biological function of Grb2 in trematodes. By bioinformatic searching of the *C. sinensis* transcriptome database, a cDNA clone (CsGrb2) encoding a putative polypeptide homologous with Grb2 was identified and retrieved. In the present study, we describe the molecular character of CsGrb2 and its possible function in *C. sinensis* organogenesis and development.

## Materials and Methods

### Ethics statement

BALB/c mice (female, 7-week-old) and rabbits (New Zealand White, male, 2.2–2.5 kg) were handled in an accredited Chung-Ang University animal facility (Accredited Unit, Korea FDA; Unit Number 36). Approval for animal experiments was obtained from Institutional Animal Care and Use Committee of Chung-Ang University (Permit Number: CAU-2011-0052 and CAU-2011-0053). This study was carried out in strict accordance with the recommendations in the Guide for the Care and Use of Laboratory Animals of the US National Institutes of Health.

### Bioinformatic analysis of CsGrb2 cDNA sequence and phylogenetic tree

The *C. sinensis* transcriptome database was analyzed using bioinformatics tools and found an EST clone encoding a polypeptide homologous with of Grb2 of other aninals. The EST clone (CSA03475) was retrieved from *C. sinensis* transcriptome glycerol stock [10] and sequenced to reconfirm to encode the putative polypeptide deduced previously. The putative amino acid sequence was predicted using ExPASy translate tool with six reading frames (http://web.expasy.org/translate/). Homologues of CsGrb2 were searched in NCBI using BLASTx. Grb2s of nematodes, insects and other vertebrate animals were retrieved from GenBank. Sequence alignment was done by comparing CsGrb2 with Grb2s of other animals and functional domains of the CsGrb2 were determined using Clustal X [11] and manually edited in GeneDoc. The tertiary structure of CsGrb2 was predicted using Phyre2 [12]. Phylogenetic tree was constructed as of Neighbor-Joining format with CsGrb2 and 18 selected Grb2s using Mega 5.2 software [13], [14]. Bootstrap value was estimated as of 1000 replicates.

### Expression and purification of recombinant full-length CsGrb2 protein

A cDNA region corresponding to the CsGrb2 open reading frame was amplified by polymerase chain reaction (PCR) using *C. sinensis* cDNA as a template. Specific forward (5′-CATATGTAGTCGACATGGAAGCTGTGGCAGTTC-3′) and reverse (5′-GTATATCAGCGGCCGCTCATGTAGCACGTGATGGATG-3′) primers containing *Sal*I or *Not*I restriction enzyme site were designed. Amplification was conducted at 95°C for 5 min followed by 30 amplification cycles (95°C for 1 min, 65°C for 30 sec, 72°C for 2 min) and a final extention at 72°C for 10 min. PCR products were purified and digested with the restriction enzymes *Sal*I and *Not*I, and then subcloned into prokaryotic expression vector pGEX-4T-3. Sequencing of the recombinant plasmid was done and revealed the insert cDNA CsGrb2 was in frame to the tag protein. After transforming *E. coli* BL21 (DE3) pLysS (Invitrogen, Carlsbad, CA, USA), sj26GST-CsGrb2 fusion protein was induced by isopropyl β-D-thiogalactopyranoside (IPTG) at a final concentration of 1 mM at 37°C for 4 hours. *E. coli* cells were harvested and lyzed by sonication. Lysates were treated with 2 M urea to solubilize insoluble recombinant protein and dialyzed against 1× phosphate buffered saline. Recombinant fusion protein was absorbed to GSH Sepharose 4B (GE Healthcare, Uppsala, Sweden) column and CsGrb2 was cleaved from sj26GST tag protein with thrombin overnight at 4°C. The cleaved CsGrb2 was eluted with PBS and subjected to SDS-polyacrylamide gel electrophoresis (SDS-PAGE). The purified target protein concentration was measured using Bio-Rad protein Assay (Bio-Rad, Philadelphia, USA).

### Production of polyclonal antibody against CsGrb2

BALB/c mice, female, 7-week-old, were purchased from Samtako BioKorea Inc. (Osan, Seoul, Korea). Recombinant CsGrb2 protein (50 µg per mouse) was emulsified with Freud's complete adjuvant and injected into mouse intraperitoneally. Two weeks later, mice were injected intraperitoneally with a booster dose of 50 µg recombinant protein emulsified with Freud's incomplete adjuvant. Two weeks after this second injection, the mice were boosted with 20 µg of recombinant protein through tail vein. Anti-CsGrb2 mouse sera were obtained one week after this third immunization.

### Animal infection and recovery of *C. sinensis* adult worms

*C. sinensis* metacercariae were collected from fresh water fish *Pseudorasbora parva* (Shen yang, China) by artificial digestion. Rabbits, New Zealand, were infected orally with the *C. sinensis* metacercariae and the adult flukes were recovered from the bile duct 6 weeks after the infection. The adult flukes were washed using 1× PBS 3 times and kept in −80°C freezer until use.

### Identification of native CsGrb2 from *C. sinensis* adult crude extract by western blotting

*C. sinensis* adult worms were washed using 50 mM PBS containing 1% Triton X-100 and 1× Complete™ mini 3 times and homogenized in 2 volumes of PBS/Complete™ mini then kept at 4°C overnight. The suspension was centrifuged at 1500 rpm for 5 min at 4°C. The supernatant was saved and centrifuged at 13000 rpm for 60 min at 4°C. The second supernatant was used as the cytosolic crude extract of *C. sinensis* adults. Concentration of the extract was determined by Bio-Rad protein assay (Bio-Rad, Philadelphia, USA).

Purified recombinant CsGrb2 protein and *C. sinensis* adult cytosolic extract were electrophoresed in 10% SDS-PAGE and then transferred onto a nitrocellulose filter (NC filter). The membrane was blocked within 5% skim milk at room temperature for 1 hour and then incubated with anti-CsGrb2 mouse serum or negative control mouse serum at a dilution of 1∶250 at 4°C overnight. After washing 3 times with PBS-T, the membrane was incubated with goat anti-mouse IgG alkaline phosphatase (AP)-conjugated antibody (Sigma, St. Louis, USA) at a dilution of 1∶5000 at room temperature for 2 hours. After washing, target protein bands were visualized using a substrate BCIP/NBT (Sigma, St. Louis, USA).

### Immunolocalization of CsGrb2 in *C. sinensis*

The *C. sinensis* in experimental rabbit liver and the metacercariae were fixed in 10% paraformaldehyde, embedded in paraffin and sectioned into ribbons. The ribbons were deparaffinized, rehydrated, and then incubated with anti-CsGrb2 mouse sera or naive mouse sera at a dilution of 1∶300 for the adult flukes or 1∶1000 for the metacercariae for 2 hours at room temperature. Subsequently, the ribbons were incubated with polymer-horse radish peroxidase (HRP) labeled anti-mouse IgG antibody (Dako Cytomation, Glostrup, Denmark). Color was developed using 3-amino-9-ethylcarbazole (AEC) as substrate and the ribbons were counterstained with hematoxylin.

### Quantitative analysis of developmental expression of CsGrb2 mRNA

The frozen *C. sinensis* adult and metacercariae were ground into powder in liquid nitrogen. Total RNA was extracted using TRIzol reagent (Ambion, California, USA) according to the manufacturer's instruction. To remove trace of genomic DNA, total RNA preparation was treated with DNA-free kit (Ambion, California, USA). Quality of RNA sample was analyzed by measuring OD_260/280_ using a spectrophotometer (Ultrospec 3000, Pharmacia Biotech, Amersham, UK). Its OD_260/280_ ratio was above 1.9. First-strand cDNA was synthesized on the total RNA with oligo-d(T) primer using Power cDNA Synthesis kit (iNtRON Biotechnology, Gyeonggi-do, Korea) according to manusfacturer's instruction.

A developmental transcription level of CsGrb2 between *C. sinensis* adults and metacercariae was measured by real time-PCR (qRT-PCR). Primers were designed using the Oligo 6 programme. For more stable comparison, three genes of *β-actin*, *calcyphosine* and *phosphoglycerate kinase* were employed as reference genes [15]. An equal amount of total cDNA, 70 ng, of the adults and metacercariae and primers were mixed with 1 µl of 10× the master mix (FasStart SYBR Green I Kit, Roche, Mannheim, Germany), making a final volume 10 µl. Thermal cycle was performed using LightCycler 1.5 (Roche, Mannheim, Germany) as follow; at first step the reaction mix was heated to 95°C for 15 min, then followed by 45 cycles of 95°C for 10 sec, 60°C for 10 sec and 72°C for 30 sec. To plot a melting curve, the PCR products were heated to 95°C for 10 sec then cooled down to 65°C over 60 sec with increment 0.1°C/sec to 95°C. Data were analyzed using LightCycler program and transcription level was calculated using 2^−ΔΔCt^ method [16].

### Enzyme-linked immunosorbent assay on recombinant CsGrb2

*C. sinensis* crude antigen and recombinant CsGrb2 were diluted in carbonate buffer to 5 µg/ml and 2 µg/ml, respectively. The antigen was aliquoted into a 96 well-plate at 200 µl per well and incubated at 4°C overnight. After washing 5 times with PBS containing 0.1% Tween 20, the plate was incubated with platyhelminth-infected patients' sera or normal human sera (for crude antigen, 24 *C. sinensis*-infected and 24 normal human sera; for recombinant CsGrb2, 44 *C. sinensis*-infected, 10 *Paragonimus westermani*-infected, 10 *Schistosoma japonicum*–infected and 10 cysticercus cellulose-infected human sera, and 40 normal human sera) for 1 hour at 37°C. Secondary antibody (horseradish peroxidase-conjugated Goat anti-human IgG antibody (Sigma, St. Louis, USA) was added to the plate and incubated at 37°C for 1 hour. Substrate o-phenylenediamine was added to the plate and color development was achieved adding 8N H_2_SO_4_ solution. ODs were measured at 490 nm using microplate reader (Molecular Devices, California, USA). The experiment was repeated 3 times.

## Results

### Molecular characters of CsGrb2

**Figure 1:**
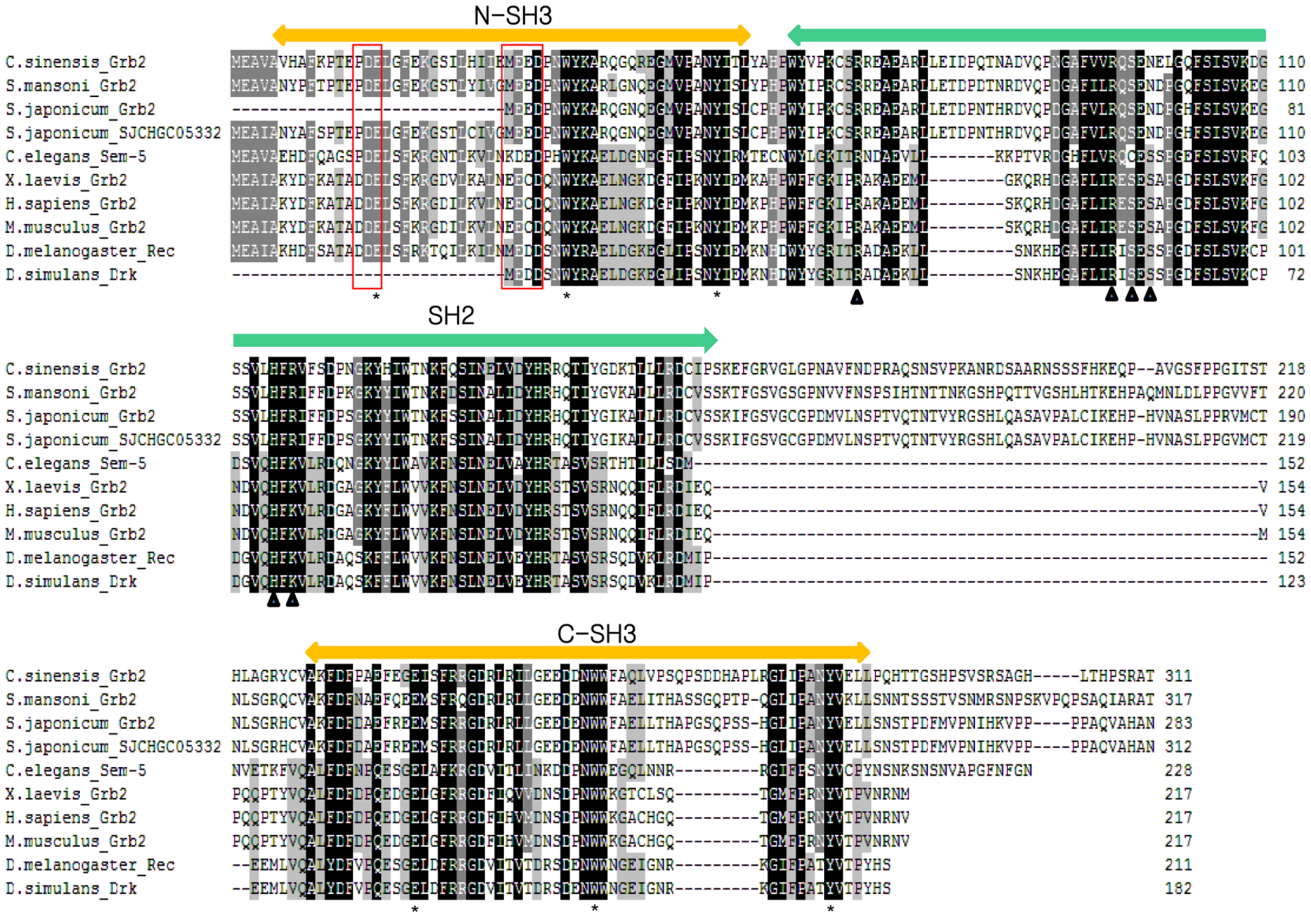
Multiple alignment of *C. sinensis* growth factor receptor-bound protein with the Grb2s of other animals. The conservation of amino acid residues are displayed with different ranks: white letters with black or gray background, black letters with gray or no background denote conservation from highest to lowest. SH3 and SH2 domains are showed in regions of bars with orange or green colors respectively. Asterisk in the SH3 domains indicates conserved residue for binding to proline-rich proteins. Triangle indicates in SH2 domain a residue conserved for binding to phosphorylated tyrosine. Red boxed residues are loops with negative charge. Aligned sequences are *Schistosoma mansoni* (XP_002575772), *Schistosoma japonicum* (CAX74268*), Schistosoma japonicum* (AAW26772), *Caenorhabditis elegans* (CCD63850.1), *Xenopus laevis* (CAB59279.1), *Homo sapiens* (CAG46740.1), *Mus musculus* (CAM21966.1), *Drosophila melanogaster* (AAN17585), *Drosophila simulans*, (EDX06936.1).

**Figure 2:**
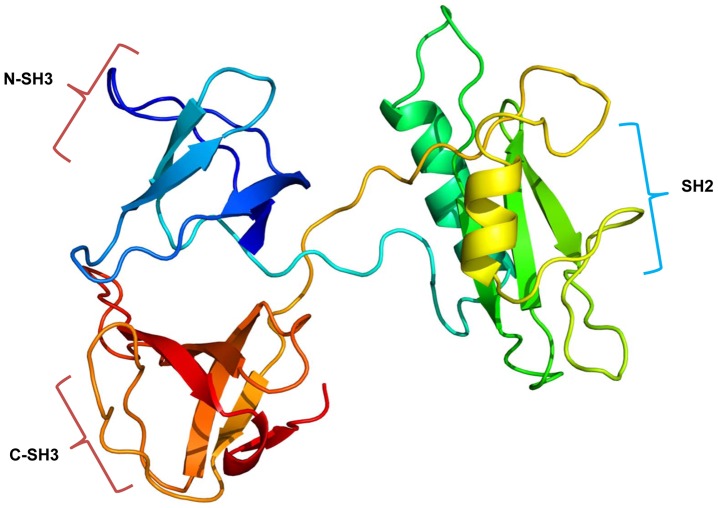
Putative three dimensional structure of *C. sinensis* growth factor receptor-bound protein (CsGrb2). The tertiary structure of CsGrb2 was predicted by online program of Phyre2. N- and C-SH3s are N- and C-terminal SH3 domains, respectively. The secondary structure of CsGrb2 was predicted to have α-helices and β-sheets.

**Figure 3:**
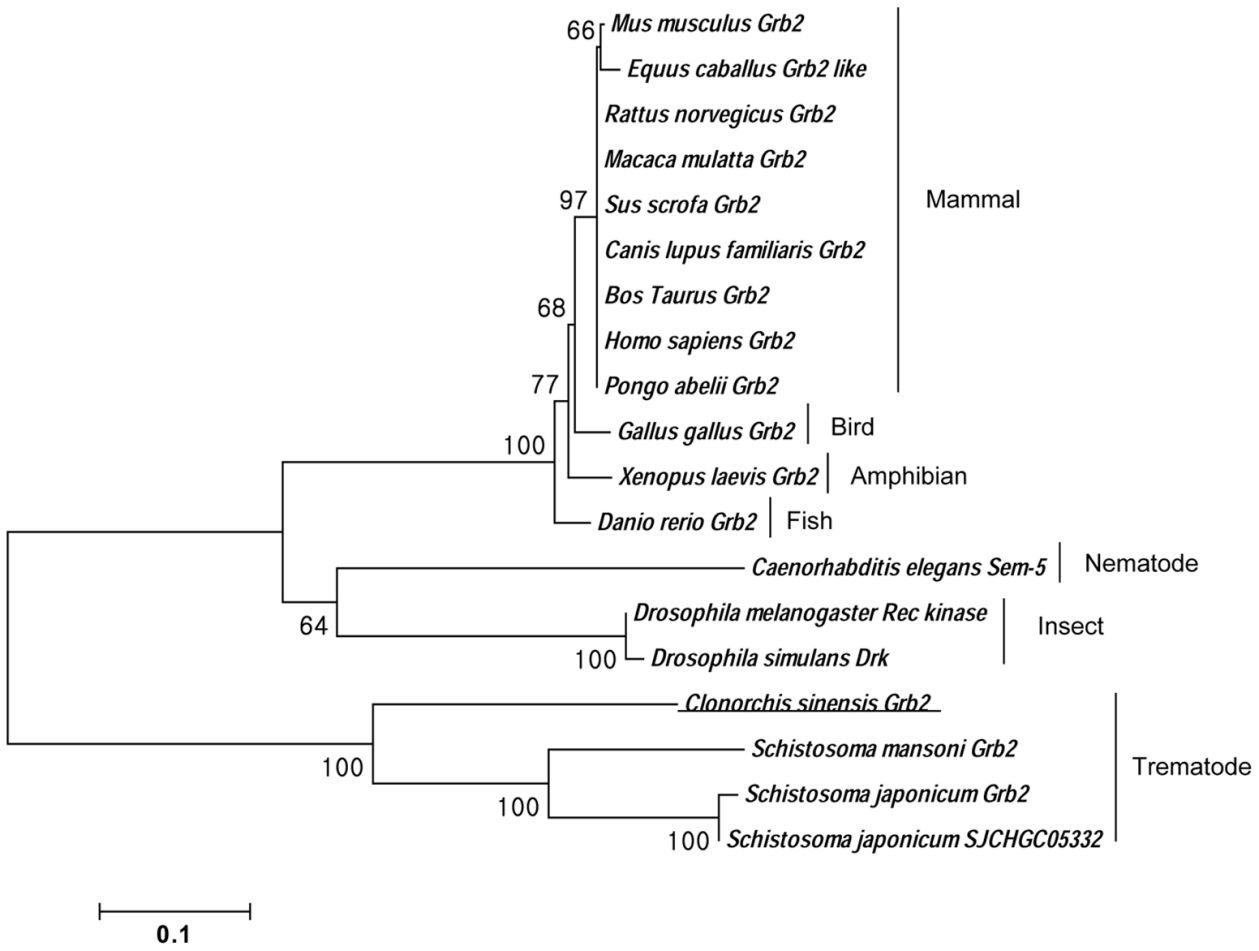
A phylogenetic tree of *C. sinensis* growth factor receptor-bound protein with those of other animals. Neighbor-Joining tree was constructed based on amino sequences of CsGrb2 and 18 Grb2 of vertebrates and invertebrates which were retrieved from GenBank. Number at each node shows bootstrap value (1000 replicates). (CAM21966.1, AEB61246, EDM06605, AFI36901, NP_001131100, XP_540431.2, DAA18091.1, CAG46740.1, NP_001126954, AAA16318, CAB59279.1, NP_998200, CCD63850.1, AAN17585, EDX06936.1, XP_002575772, CAX74268, and AAW26772) is GenBank accession number.

A cDNA clone (CSA03475) retrieved from the *C. sinensis* transcriptome encoded a polypeptide of 311 amino acids, showing high sequence identity with Grb2 of invertebrates and vertebrate animals (Figure 1). By comparing with the secondary structure of Grb2 in other organisms, CsGrb2 was found to have one SH2 domain (with 5 conserved Grb2 motifs) flanked by two SH3 domains [17]. The residues in SH2 and SH3 domains for binding target molecules were highly conserved. A conceptual tertiary structure of this polypeptide showed that N-terminal SH3 domain had 4 β-sheets, and C-terminal SH3 domain had 5 β-sheets, and SH2 domain consisted of 5-βsheets and 2 α-helices. CsGrb2 showed a very long loop between the SH2 and N-ternimal SH3 domains, which also existed in *Schistosoma mansoni* and *Schistosoma japonicum* Grb2s, but not in the other Grb2s (Figures 1 & 2). In the SH2 domain, residues binding to the phosphorylated tyrosine were highly conserved. In the N- and C-terminal SH3 domains, residues bingding to the prolin-rich proteins were highly conserved at the identical position to other Grb2s. The first and second loops on the C-terminal SH3 domain had several conserved residues of negative charge. Given this bioinformatics results, this cDNA clone was considered to encode a growth factor receptor bound protein of *C. sinensis* (CsGrb2; Fig. 1). A phylogenetic analysis showed that CsGrb2 was grouped with homologs of trematodes such as *Schistosoma mansoni* and *Schistosoma japonicum* (Figure 3). The mammalian Grb2s formed a major clade and the vertebrates the second. The insect Grb2s grouped a clade from others.

### Recombinant and native CsGrb2 proteins

**Figure 4:**
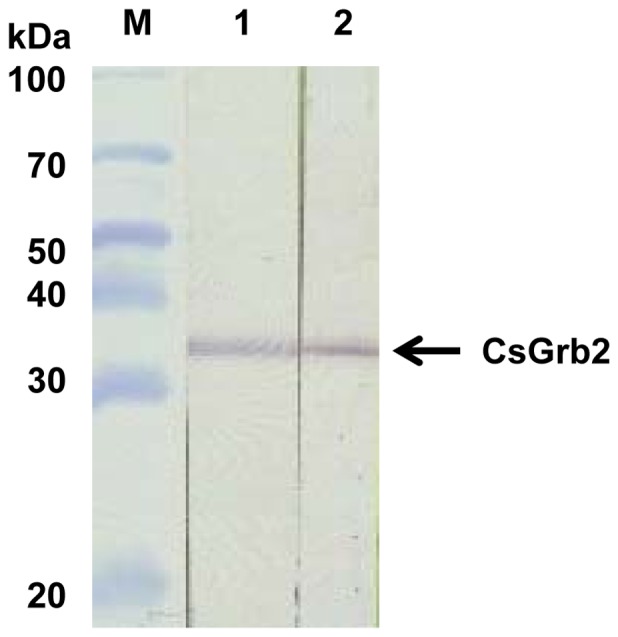
Immunoblotting of recombinant and native *C. sinensis* growth factor receptor-bound proteins (CsGrb2). Blots were probed with CsGrb2-immune mouse serum. Lane 1, recombinant CsGrb2; Lane 2, crude extract of adult *C. sinensis*. M, protein molecular weight marker.

Recombinant fusion protein Sj26GST-CsGrb2 (60 kDa) was induced to be expressed in *E. coli* BL21 (DE3) pLysS (Figure S1). The *E. coli* were pelletted by spinning and lysed in 2 M urea to solubilize the fusion protein. After dialysis against PBS for 2 hours, the fusion protein bound efficiently to GSH-Sepharose beads. Recombinant CsGrb2 protein (34 kDa) was claved from its tag-protein Sj26GST by thrombin treatment, the eluted at high purity with PBS (Figure S1). This purified recombinant CsGrb2 protein was used for immune sera production from mice. The CsGrb2 immune mouse sera recognized native CsGrb2 from *C. sinensis* soluble extract (Figure 4) showing an estimated molecular mass of its recombinant form, 34 kDa.

### Developmental distribution of CsGrb2 in the *C. sinensis* adult and metacercaria

**Figure 5:**
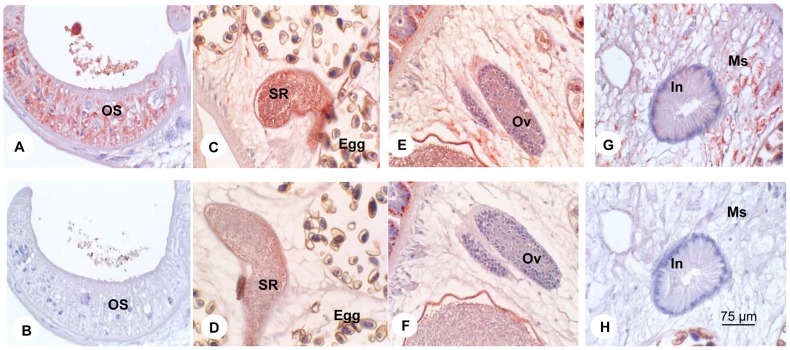
Localization of growth factor receptor-bound protein (CsGrb2) in *C. sinensis* adult. Panels A and B, oral sucker (OS). Panels C and D, seminal receptacle (SR); Panels E and F, ovary (Ov); Panels G and H, intestine (In) in mesenchymal tissue (Ms). Panels A, C, E and G were treated with anti-CsGrb2 mouse serum, and Panels B, D, F and H with normal mouse serum. Original magnification, ×400.

**Figure 6:**
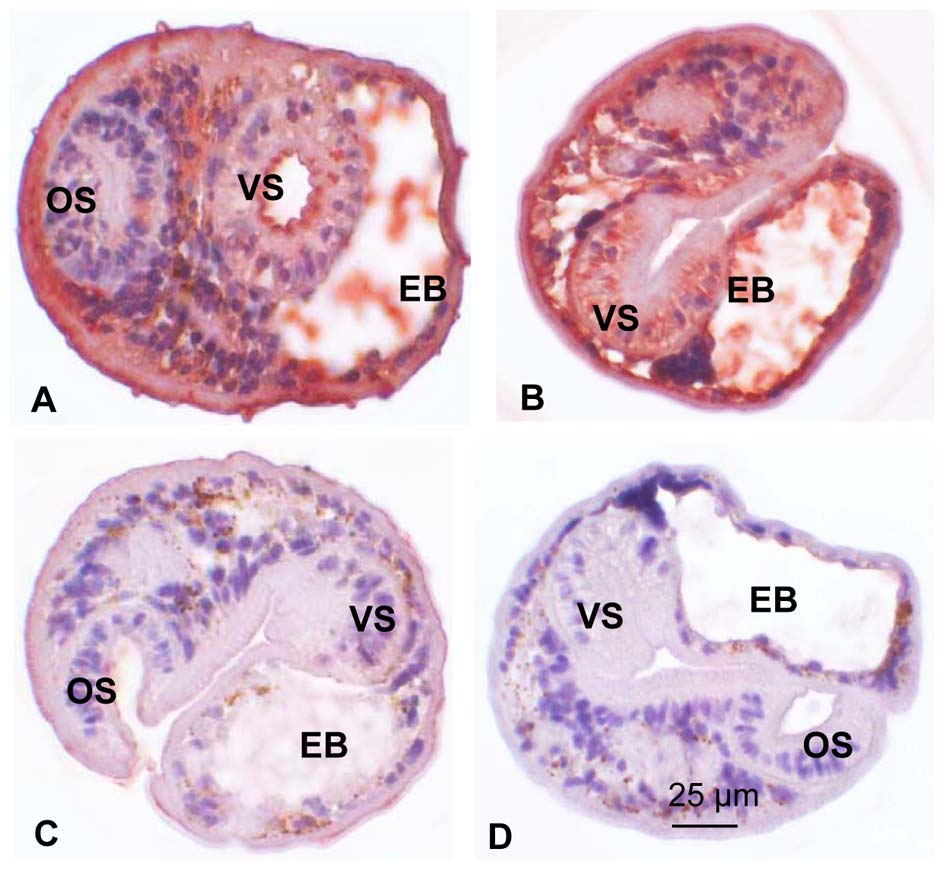
Localization of growth factor receptor-bound protein (CsGrb2) in *C. sinensis* metacercariae. Metacercariae were treated with anti-CsGrb2 mouse serum (A and B) or with normal mouse serum (C) at a dilution of 1∶1000 or no serum (D). OS, oral sucker; VS, ventral sucker; EB, excretory bladder.

The CsGrb2 immune sera were used for immunohistochemical localization of CsGrb2 *in C. sinensis* adult worms and metacercariae. In the adult worms, strong color staining was found in the oral sucker and followed by the sperms in the seminal receptacle, the mesenchymal tissues, the intra-uterine eggs and the ovary (Figure 5). Weak staining was observed in the testes and the vitelline follicles (data not shown). In the metacercariae, color staining much stronger than in the adults, was observed in the tegument, subtegument, mesenchymal tissues, and the musculature of the oral and ventral suckers (Figure 6).

### Expression of CsGrb2 in the developmental stages

**Figure 7:**
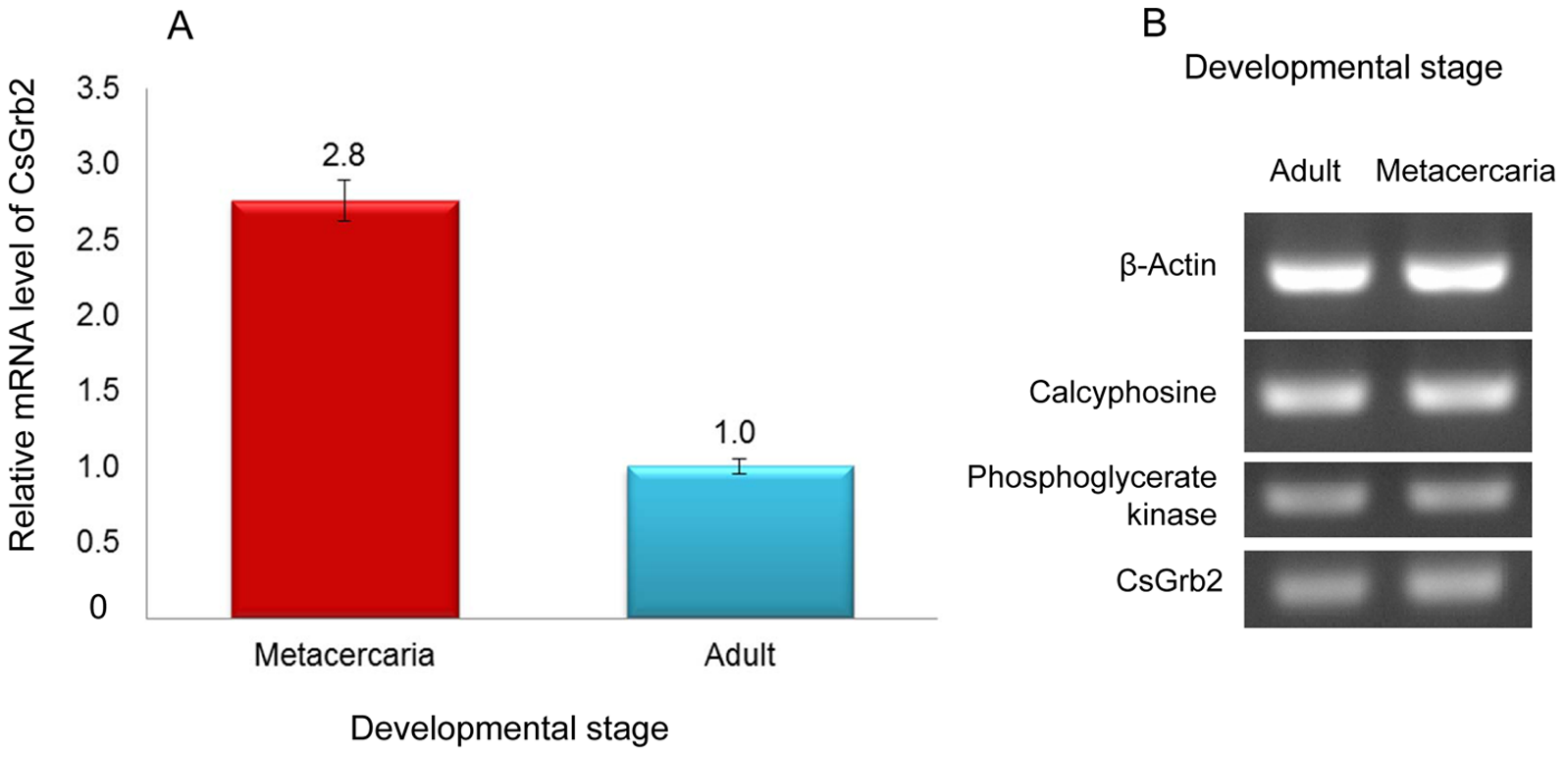
Relative transcriptional level of mRNA of *C. sinensis* growth factor receptor-bound protein in the developmental stages. Quantitative RT-PCR was performed on adult and metacercarial mRNA. A, relative mRNA level of *C. sinensis* adults and metacercariae. For quantification, refer to Materials and Methods; B, agarose gel electrophoresis of amplication products of quantitative RT-PCR.

Developmental expression of the CsGrb2 in the *C. sinensis* adults and metacercariae was analyzed by quantitative real time PCR using total RNA as template. A relative transcription level of the target gene CsGrb2 was from C_T_-values of target and reference genes using 2^−ΔΔCt^ method [16]. The CsGrb2 level was 2.8 times higher in the metacercariae than in the adults (Figure 7A). To check PCR-amplification and relative quantification, PCR product were electrophorosed in an agarsoe gel. The amplified DNA bands of three reference genes [10] showed equal thickness in the two stages. Referred to the reference gene products, the amplified DNA band of the CsGrb2 revealed thicker in the metacercariae than in the adults (Figure 7B), supporting the relative quantification by qRT-PCR and 2^−ΔΔCt^ method [16].

### Recombinant CsGrb2 protein as serodiagnostic antigen

**Figure 8:**
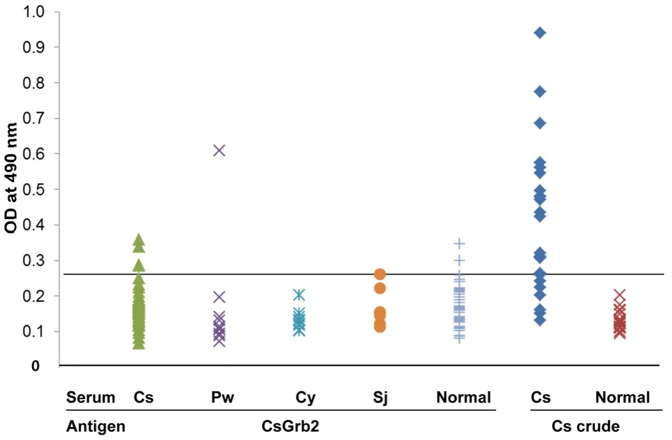
Antigenicity of recombinant *C. sinensis* growth factor receptor-bound protein towards IgG antibody in *C. sinensis* and helminth-infected human sera by ELISA. Cs, clonorchiasis sinensis; Pw, paragonimiasis westermani; Cy, cysticercosis cellulosae; Sj, schistosomiasis japonicum; Normal, uninfected human sera were used as a negative control.

Antigenicity of the recombinant CsGrb2 protein was evaluated by ELISA using human sera infected with platyhelminths. The *C. sinensis* adult soluble extract was employed as a positive control. A cutoff of OD_490_ value was set at 0.29, which is an average +3SD of OD values of the normal control sera. ELISA results showed that recombinant CsGrb2 protein had low sensitivity to *C. sinensis*-infected sera but high specificity for platyhelminth-infected human sera, compared to the *C. sinensis* soluble antigen (Figure 8).

## Discussion

Growth factor receptor bound protein 2 (Grb2) has been shown to play an important role in Ras signal transduction pathways. In the present study, CsGrb2 showed domain similarity with Grb2 of human and animals. Motifs consisting of residues RRSSHK are conserved in the SH2 domains of different organisms, and form a hydrophobic phosphotyrosine binding pocket [18]. In CsGrb2, the second S and K are replaced by N and R respectively, indicating that some residues have species specificity. However, motif RRXXHX is consercved in all SH2 domains of Grb2. The SH2 domain binds to phosphotyrosines in growth factor receptors by recognizing an YXNX motif [19], [20]. However, a repetitive peptide G1 of 11 amino acids with the same YXNX sequence, without phosphotyrosine, had been reported to bind to the SH2 domain of Grb2 [21], suggesting that Grb2 participates in distinct functional pathways under stimulation of various ligands in different cell types.

The structures of the N-terminal and C-terminal SH3 domains showed some different feature. Both SH3 domains have the RGD sequence that forms an accessible turn present in mammalian Grb2. The RGD of N-SH3 domain was replaced by KGS in *C. sinensis*, by RGN in *Caenorhabditis elegans* and by KTQ in *Drosophila melanogaster* [22], [23]. The SH3 domains are separated from the SH2 domain by a bridge, like YAHP in *C. sinensis* or KPHP in others, suggesting that a conformational change in the SH2 domain has no effect on SH3 binding to proline rich proteins [24], [25]. The N-SH3 domain is nearer to the SH2 domain than the C-SH3 domain is. In Grb2s of the trematodes, the loop between the SH2 and C-SH3 domains was found much longer than in other Grb2s, rendering more flexibility to the domains. The SH3 domains bind to a proline rich motif in the C-terminus of Sos, VPVPPPVPPRRRP, especially to PPPVPPR [26], [27], [28], [29], and the binding affinity of N-SH3 to proline rich motif is ten times higher than that of C-SH3 [24].

In *C. sinensis*, CsGrb2 was expressed higher in the metacercairae than in the adults and showed an extensive distribution in the metacercariae. The metacercariae has exclusively somatic organs such as oral and ventral suckers, pharynx, esophagus and excretory bladder. A genital anlage is present as an indistinctive rudiment posterior to the ventral sucker. During development to adult *C. sinensis*, the anlage differentiates to reproductive organs such as ovary, vitelline glands, uterus, seminal receptacle, Mehlis' gland, testes and seminal vesicle. The high expression and wide distribution of CsGrb2 in the metacercariae may imply that it is required for development of *C. sinensis* from the metacercariae to the adults. As an adptor, Grb2/Sem-5 is closely related to formation of vulva in *C. elegans* [22] and to organogenesis in mouse [30]. In *Drosophilia*, Drk, a homologue of Grb2, is necessary for Sos-mediated Ras signal pathway leading a development of precursor cells to the eyes [23].

In adult *C. sinensis*, CsGrb2 was abundantly distributed in the somatic and reproductive organs, suggesting that CsGrb2 may have functions in these tissues. Adult *C. sinensis* produce a large mount of eggs every day through mitogenesis and meiosis. Grb2 is highly expressed as an adaptor in signal transduction pathway leading to mitogenesis and found to reinitiate meiosis in *Xenopus* oocytes [31]. In rat, Ash/Grb2 also links receptor PTKs to Ras, Rac and Rho which belong to the same family and lead to mitogenesis, membrane ruffle formation [32]. Besides the Ras signaling pathway, Grb2 binds to dynamin, a protein essential for endocytosis, synaptic transmission and neurogenesis, through its SH3 domain [33], [34]. It is, therefore, suggested that CsGrb2 may be involved in meiosis during spermatogenesis and ovogenesis of the adult *C. sinensis*.

Oral sucker is a very important locomotorium of *C. sinensis* with lots of myocytes. *C. sinensis* can move depending on strong hold of the oral and ventral suckers and extension of the forebody. In present study, CsGrb2 was found abundant in the oral sucker, suggesting that CsGrb2 may play a role also in this tissue. In mice, Grb2 is necessary for migratory myoblasts and secondary fiber development by binding to Met receptor under the stimulation of hepatocyte growth factor [35], [36]. To keep muscle cell viability, dystroglycan, a novel laminin receptor, interacts with Grb2 to link the extracellular matrix and sarcolemma in skeletal muscle [37]. In points of view, it is suggested that CsGrb2 may play a role for muscle development and action in the *C. sinensis*.

CsGrb2 was also found to be widely expressed in the mesenchymal tissues that composed of many adipocytes in *C. sinensis* adults. In rats and mice, Grb2 is widely distributed in many organs [29], [38], suggesting that Grb2 was also important for basic metabolism [39]. Grb2 has been found to bind to tyrosine-phosphorylated insulin receptor substrate-1, leading to the stimulation of DNA synthesis [40], increased expression and activity of the GLUT-1 glucose transporter [41]. GLUT-1 is present in many tissues and responsible for basal glucose uptake. In adipocytes, IRS-1/Grb2 also plays an important role in insulin-induced glucose transport and mitogenesis [42], [43], [44]. Glucose absorption and metabolism is critical for *C. sinensis* since it lives in an oxygen deficient environment [2]. Glucose transporters and sodium/glucose co-transporters were found to be expressed at high levels in the adult *C. sinensis* [10]. Glycolytic enzymes were also of the most abundantly expressed genes in the *C. sinensis* developmental transcriptome [10]. Based on these findings, we suggest that CsGrb2 may play a regulatory function during energy metabolism of *C. sinensis*, which deserves further investigation.

For low antibody titers were elicited in *C. sinensis* infected people, antigenicity of recombinant CsGrb2 was not significant. As a serodiagnostic antigen, therefor the CsGrb2 recombinant protein seems less useful since this protein revealed a low sensitivity.

In conclusion, CsGrb2 was cloned, produced in recombinant protein and its biological characters were analyzed. The CsGrb2 may play roles in development, organogenesis, glucose transport and lipolytic metabolism of *C. sinensis*. The CsGrb2 deserves further studies to understand its molecular biological functions and to comprehensively widely parasitic and free-living platyhelminths.

## Supporting Information

Figure S1 **Purification and on-bead cleavage of recombinant** ***C. sinensis*** **growth factor receptor-bound protein.** Proteins were deployed by 10% SDS-PAGE. *E. coli* BL21 (DE3) pLysS were transformed with expression plasmid construct pGEX-4T-CsGrb2. Lane 1, uninduced *E. coli* lysate; Lane 2, soluble fraction of induced *E. coli* lysate; Lanes 3–4, first and second eluates after thrombin cleavage; M, protein molecular weight marker.
(TIF)

## References

[1] Lun ZR, Gasser RB, Lai DH, Li AX, Zhu XQ, et al (2005) Clonorchiasis: a key food-borne zoonosis in China. Lancet Infect Dis 5: 31–41. 15620559 10.1016/S1473-3099(04)01252-6

[2] Kim TI, Yoo WG, Kwak BK, Seok JW, Hong SJ (2011) Tracing of the Bile-chemotactic migration of juvenile *Clonorchis sinensis* in rabbits by PET-CT. PLoS Negl Trop Dis 5: e1414. 22180795 10.1371/journal.pntd.0001414 PMC3236719

[3] Shin HR, Oh JK, Masuyer E, Curado MP, Bouvard V, et al (2010) Epidemiology of cholangiocarcinoma: an update focusing on risk factors. Cancer Sci 101: 579–585. 20085587 10.1111/j.1349-7006.2009.01458.x PMC11158235

[4] Buday L, Downward J (1993) Epidermal growth factor regulates p21ras through the formation of a complex of receptor, Grb2 adapter protein, and Sos nucleotide exchange factor. Cell 73: 611–620. 8490966 10.1016/0092-8674(93)90146-h

[5] Vidal M, Montiel JL, Cussac D, Cornille F, Duchesne M, et al (1998) Differential interactions of the growth factor receptor-bound protein 2 N-SH3 domain with son of sevenless and dynamin. J Biol Chem 273: 5343–5348. 9478994 10.1074/jbc.273.9.5343

[6] Bar-Sagi D, Rotin D, Batzer A, Mandiyan V, Schlessinger J (1993) SH3 domains direct cellular localization of signaling molecules. Cell 74: 83–91. 8334708 10.1016/0092-8674(93)90296-3

[7] Yu GZ, Chen Y, Wang JJ (2009) Overexpression of Grb2/HER2 signaling in Chinese gastric cancer: their relationship with clinicopathological parameters and prognostic significance. J Cancer Res Clin Oncol 135: 1331–1339. 19337752 10.1007/s00432-009-0574-8 PMC12160245

[8] Yoon SY, Jeong MJ, Yoo J, Lee KI, Kwon BM, et al (2002) Grb2 dominantly associates with dynamin II in human hepatocellular carcinoma HepG2 cells. J Cell Biochem 84: 150–155. 10.1002/jcb.1275 11746524

[9] Kondo A, Hirayama N, Sugito Y, Shono M, Tanaka T, et al (2008) Coupling of Grb2 to Gab1 mediates hepatocyte growth factor-induced high intensity ERK signal required for inhibition of HepG2 hepatoma cell proliferation. J Biol Chem 283: 1428–1436. 18003605 10.1074/jbc.M704999200

[10] Yoo WG, Kim DW, Ju JW, Cho PY, Kim TI, et al (2011) Developmental transcriptomic features of the carcinogenic liver fluke, *Clonorchis sinensis* . PLoS Negl Trop Dis 5: e1208. 21738807 10.1371/journal.pntd.0001208 PMC3125140

[11] Larkin MA, Blackshields G, Brown NP, Chenna R, McGettigan PA, et al (2007) Clustal W and Clustal X version 2.0. Bioinformatics 23: 2947–2948. 17846036 10.1093/bioinformatics/btm404

[12] Nema V, Pal SK (2013) Exploration of freely available web-interfaces for comparative homology modelling of microbial proteins. Bioinformation 9: 796–801. 24023424 10.6026/97320630009796 PMC3766314

[13] Tamura K, Peterson D, Peterson N, Stecher G, Nei M, et al (2011) MEGA5: molecular evolutionary genetics analysis using maximum likelihood, evolutionary distance, and maximum parsimony methods. Mol Biol Evol 28: 2731–2739. 21546353 10.1093/molbev/msr121 PMC3203626

[14] Hall BG (2013) Building phylogenetic trees from molecular data with MEGA. Mol Biol Evol 30: 1229–1235. 23486614 10.1093/molbev/mst012

[15] Yoo WG, Kim TI, Li S, Kwon OS, Cho PY, et al (2009) Reference genes for quantitative analysis on *Clonorchis sinensis* gene expression by real-time PCR. Parasitol Res 104: 321–328. 18815810 10.1007/s00436-008-1195-x

[16] Livak KJ, Schmittgen TD (2001) Analysis of relative gene expression data using real-time quantitative PCR and the 2^−ΔΔCt^ method. Methods 25: 402–408. 11846609 10.1006/meth.2001.1262

[17] Koch CA, Anderson D, Moran MF, Ellis C, Pawson T (1991) SH2 and SH3 domains: elements that contral interactions of cytoplasmic signaling proteins. Science 252: 668–674. 1708916 10.1126/science.1708916

[18] Chardin P, Cussac D, Maignan S, Ducruix A (1995) The Grb2 adaptor. FEBS Lett 369: 47–51. 7641883 10.1016/0014-5793(95)00578-w

[19] Denkinger DJ, Borges CR, Butler CL, Cushman AM, Kawahara RS (2000) Genomic organization and regulation of the *vav* proto-oncogene. Biochim Biophys Acta 1491: 253–262. 10760587 10.1016/s0167-4781(00)00008-7

[20] Songyang Z, Shoelson SE, Chaudhuri M, Gish G, Pawson T, et al (1993) SH2 domains recognize specific phospeptide sequences. Cell 72: 767–778. 7680959 10.1016/0092-8674(93)90404-e

[21] Oligino L, Lung FD, Sastry L, Bigelow J, Cao T, et al (1997) Nonphosphorylated peptide ligands for the Grb2 Src homology 2 domain. J Biol Chem 272: 29046–29052. 9360978 10.1074/jbc.272.46.29046

[22] Clark SG, Stern MJ, Horvitz HR (1992) *C. elegans* cell-signalling gene sem-5 encodes a protein with SH2 and SH3 domains. Nature 356: 340–344. 1372395 10.1038/356340a0

[23] Olivier JP, Raabe T, Henkemeyer M, Dickson B, Mbamalu G, et al (1993) A *Drosophila* SH2-SH3 adaptor protein implicated in coupling the sevenless tyrosine kinase to an activator of Ras guanine nucleotide exchange, Sos. Cell 73: 179–191. 8462098 10.1016/0092-8674(93)90170-u

[24] Cussac D, Frech M, Chardin P (1994) Binding of the Grb2 SH2-domain to phosphotyrosine motifs does not change the affinity of its SH3-domains for Sos proline-rich motifs. EMBO J 13: 4011–4021. 7521298 10.1002/j.1460-2075.1994.tb06717.x PMC395321

[25] Maignan S, Guilloteau JP, Fromage N, Arnoux B, Becquart J, et al (1995) Crystal structure of the mammalian Grb2 adaptor. Science 268: 291–293. 7716522 10.1126/science.7716522

[26] Chardin P, Camonis JH, Gale NW, van Aelst L, Schlessinger J, et al (1993) Human Sos1: a guanine nucleotide exchange factor for Ras that binds to Grb2. Science 260: 1338–1343. 8493579 10.1126/science.8493579

[27] Egan SE, Giddings BW, Brooks MW, Buday L, Sizeland AM, et al (1993) Association of Sos-Ras exchange protein with Grb2 is implicated in tyrosine kinase sigal transduction and transformation. Nature 363: 45–51. 8479536 10.1038/363045a0

[28] Li N, Batzer A, Daly R, Yajnik V, Skolnik E, et al (1993) Guanine-nucleotide-releasing factor hSos1 binds to Grb2 and links receptor tyrosine kinases to Ras signalling. Nature 363: 85–88. 8479541 10.1038/363085a0

[29] Williamson MP (1994) The structure and function of proline-rich regions in proteins. Biochem J 297: 249–260. 8297327 10.1042/bj2970249 PMC1137821

[30] Suen KL, Bustelo XR, Pawson T, Barbacid M (1993) Molecular cloning of the mouse *grb2* gene: differential interaction of the Grb2 adaptor protein with epidermal growth factor and nerve growth factor receptors. Mol Cell Biol 13: 5500–5512. 7689150 10.1128/mcb.13.9.5500-5512.1993 PMC360265

[31] Cailliau K, Browaeys-Poly E, Broutin-L'Hermite I, Nioche P, Garbay C, et al (2001) Grb2 promotes reinitiation of meiosis in *Xenopus* oocytes. Cell Signal 13: 51–55. 11257447 10.1016/s0898-6568(00)00138-8

[32] Matuoka K, Shibasaki F, Shibata M, Takenawa T (1993) Ash/Grb-2, a SH2/SH3-containing protein, couples to signaling for mitogenesis and cytoskeletal reorganization by EGF and PDGF. EMBO J 12: 3467–3473. 8253073 10.1002/j.1460-2075.1993.tb06021.x PMC413623

[33] Vidal M, Goudreau N, Cornille F, Cussac D, Gincel E, et al (1999) Molecular and cellular analysis of Grb2 SH3 domain mutants: interaction with Sos and dynamin. J Mol Biol 290: 717–730. 10395825 10.1006/jmbi.1999.2899

[34] Scaife R, Gout I, Waterfield MD, Margolis RL (1994) Growth factor-induced binding of dynamin to signal transduction proteins involves sorting to distinct and separate proline-rich dynamin sequences. EMBO J 13: 2574–2582. 8013457 10.1002/j.1460-2075.1994.tb06547.x PMC395131

[35] Cheng AM, Saxton TM, Sakai R, Kulkarni S, Mbamalu G, et al (1998) Mammalian Grb2 regulates multiple steps in embryonic development and malignant transformation. Cell 95: 793–803. 9865697 10.1016/s0092-8674(00)81702-x

[36] Maina F, Casagranda F, Audero E, Simeone A, Comoglio PM, et al (1996) Uncoupling of Grb2 from the Met receptor *in vivo* reveals complex roles in muscle development. Cell 87: 531–542. 8898205 10.1016/s0092-8674(00)81372-0

[37] Yang B, Jung D, Motto D, Meyer J, Koretzky G, et al (1995) SH3 domain-mediated interaction of dystroglycan and Grb2. J Biol Chem 270: 11711–11714. 7744812 10.1074/jbc.270.20.11711

[38] Matuoka K, Shibata M, Yamakawa A, Takenawa T (1992) Cloning of ASH, a ubiquitous protein composed of one Src homology region (SH) 2 and two SH3 domains, from human and rat cDNA libraries. Proc Natl Acad Sci (USA) 89: 9015–9019. 1384039 10.1073/pnas.89.19.9015 PMC50055

[39] Pan Z, Wang J, Yin X, Xie P, Yang J, et al (2008) The function study on the interaction between Grb2 and AMPK. Mol Cell Biochem 307: 121–127. 17849173 10.1007/s11010-007-9591-6

[40] Skolnik EY, Batzer A, Li N, Lee CH, Lowenstein E, et al (1993) The function of GRB2 in linking the insulin receptor to Ras signaling pathways. Science 260: 1953–1955. 8316835 10.1126/science.8316835

[41] Bonini JA, Colca JR, Dailey C, White M, Hofmann C (1995) Compensatory alterations for insulin signal transduction and glucose transport in insulin-resistant diabetes. Am J Physiol 269: E759–765. 7485492 10.1152/ajpendo.1995.269.4.E759

[42] Kaburagi Y, Satoh S, Tamemoto H, Yamamoto-Honda R, Tobe K, et al (1997) Role of insulin receptor substrate-1 and pp60 in the regulation of insulin-induced glucose transport and GLUT4 translocation in primary adipocytes. J Biol Chem 272: 25839–25844. 9325314 10.1074/jbc.272.41.25839

[43] Mur C, Valverde AM, Kahn CR, Benito M (2002) Increased insulin sensitivity in IGF-I receptor−deficient brown adipocytes. Diabetes 51: 743–754. 11872675 10.2337/diabetes.51.3.743

[44] Páez-Espinosa V, Rocha EM, Velloso LA, Saad MJ (2001) Regulation of insulin-stimulated tyrosine phosphorylation of Shc and Shc/Grb2 association in liver, muscle, and adipose tissue of epinephrine- and streptozotocin-treated rats. Endocrine 14: 295–302. 11444425 10.1385/ENDO:14:3:295

