# Supplementary figures and images for: Molecular Cloning and Characterization of Growth Factor Receptor Bound-Protein in Clonorchis sinensis
Source: PLoS One. 2014 Jan 16;9(1):e85577. doi: 10.1371/journal.pone.0085577 (PMC3894193; doi:10.1371/journal.pone.0085577)

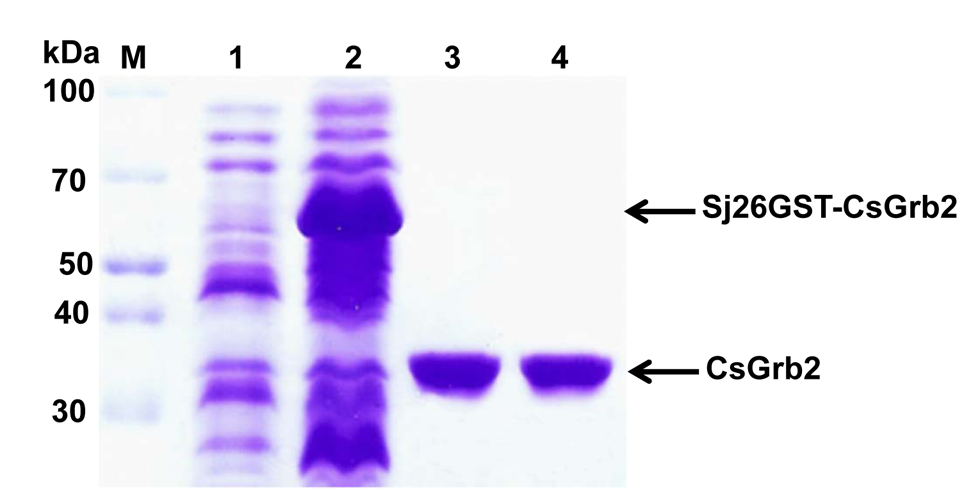

Supplement: Figure S1 — Purification and on-bead cleavage of recombinant C. sinensis growth factor receptor-bound protein. Proteins were deployed by 10% SDS-PAGE. E. coli BL21 (DE3) pLysS were transformed with expression plasmid construct pGEX-4T-CsGrb2. Lane 1, uninduced E. coli lysate; Lane 2, soluble fraction of induced E. coli lysate; Lanes 3–4, first and second eluates after thrombin cleavage; M, protein molecular weight marker. (TIF) [file pone.0085577.s001.tif]
